# High Anal Canal Pressure and Rectal Washouts Contribute to the Decrease of Anal Basal Pressure After Botulinum Toxin Injections in Paediatric Patients With Chronic Constipation

**DOI:** 10.3389/fped.2022.819529

**Published:** 2022-03-22

**Authors:** Ge Sun, Monika Trzpis, Paul M. A. Broens

**Affiliations:** ^1^Department of Surgery, Anorectal Physiology Laboratory, University of Groningen, University Medical Center Groningen, Groningen, Netherlands; ^2^Department of Surgery, Division of Pediatric Surgery, University of Groningen, University Medical Center Groningen, Groningen, Netherlands

**Keywords:** pediatric constipation, manometry, physiology, rectal washout, anal basal pressure, botulinum toxin

## Abstract

**Introduction:**

Chronic constipation can be treated by injecting botulinum toxin into the anal sphincter to decrease anal basal pressure. To assess the effect of botulinum toxin, we investigated the factors that contribute to changes in anal basal pressure after injection.

**Methods:**

This was a retrospective study conducted in a tertiary hospital in the Netherlands. We included children with chronic constipation treated with botulinum toxin injections and measured anal basal pressure before and after each injection. Multivariable linear regression analyses were used.

**Results:**

We investigated 30 cases with idiopathic constipation. Their median age was 20.5 (7.75–53.25) months. Anal basal pressure decreased after injection in 20 cases. The mean decrease of anal basal pressure after injection was 18.17 ± 35.22 mmHg. The anal basal pressure change was linearly correlated with preinjection pressure (*R*^2^ = 0.593, *P* < 0.001). A significant decrease of pressure was observed in patients with preinjection pressure > 70 mmHg. Preinjection anal basal pressure (β = −0.913, *P* < 0.001) and rectal washouts (β = −21.015, *P* = 0.007) contributed significantly to pressure changes. Changes in anal basal pressure were also significantly associated with patients' weights (β = 0.512, 95% CI, 0.011–1.013) and sex (β = 22.971, 95% CI, 9.205–36.736).

**Conclusions:**

Botulinum toxin significantly decreases anal basal pressure when preinjection pressure is higher than 70 mmHg. In patients with severely elevated anal basal pressure, we recommend rectal washouts to promote the decrease of anal basal pressure.

## Introduction

Chronic constipation is often encountered in children and can be present as idiopathic constipation or as a result of an organic problem, such as anorectal malformation or Hirschsprung's disease (1–4). Dietary fibers, laxatives and enemas are often prescribed to treat idiopathic chronic constipation (5). If necessary, these remedies are also used to support defecation in children with organic constipation who have already been treated for the organic cause. Alternatively, if a child does not respond to conservative treatment, botulinum toxin injections in the anal sphincter can be administered (2, 3, 5–9). The idea underlying botulinum toxin therapy for treating chronic constipation stems from the assumption that elevated anal basal pressure indicates chronic contraction of the anal sphincter, disabling its relaxation when emptying the rectum is appropriate and thus hampering defecation (10, 11). Indeed, there are studies that confirm the association between elevated anal basal pressure and chronic constipation (12, 13). Botulinum toxin was introduced to force the anal sphincter to relax, thereby decreasing pressure in the anal canal and thus relieving intractable constipation (14, 15). This type of treatment, however, is not effective in all constipated patients (3). In the case of children, the efficacy regarding symptom improvement varies between 17 to 91% (16). This wide range may be the result of the different methodological designs of studies and of the fact that efficacy is based primarily on symptom improvement which, in case of children and toddlers in particular, relies mostly on their parents' opinions and might therefore be subjective. Nevertheless, it is undeniable that some patients do not respond to botulinum toxin therapy. To date, no factor has been identified that significantly determines the efficacy of botulinum toxin to reduce anal basal pressure. Seeing that constipation itself is associated with demographic factors such as age and sex (17), it could be that these factors also contribute to the efficacy of botulinum toxin therapy.

Botulinum toxin therapy requires anesthesia and as such it is invasive. Therefore, there is need to find predictive factors that enable us to distinguish between patients who will respond to the treatment and those who will not; the latter should subsequently be offered a different type of treatment (18–20).

Based on our clinical experience, we hypothesize that anal basal pressure measured before injection of botulinum toxin may be one of the factors that determines the response of the anal sphincter to this neurotoxin. In this study, we aimed to investigate factors that contribute to the decrease of anal basal pressure in pediatric patients whom we treated with botulinum toxin for chronic constipation.

## Materials and Methods

### Patients and Data Collection

**Figure 1:**
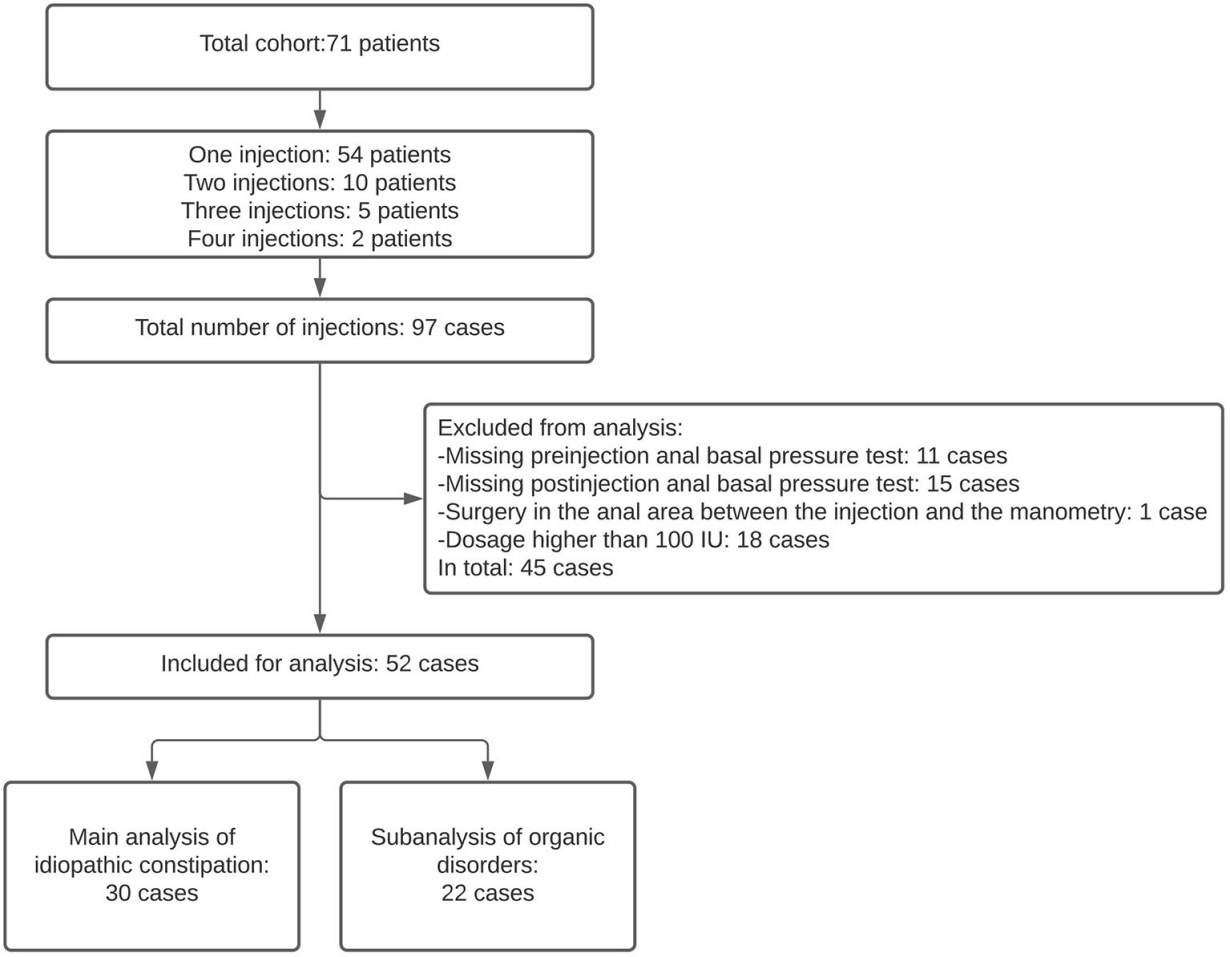
Flowchart illustrating patients' inclusion and exclusion steps.

This was a retrospective observational study. We included pediatric patients who received at least one botulinum toxin injection for severe chronic constipation and who underwent anorectal physiology tests at the Anorectal Physiology Laboratory at the University Medical Center of Groningen before and after treatment between March 2013 and November 2020. The patients were diagnosed with chronic constipation and failed to respond to conservative treatment. This included laxatives, enemas, and finally, rectal washouts if their defecation remained troublesome even after all the conservative treatments had been tried. Some patients were treated with botulinum toxin more than once (Figure 1). We defined each injection as a separate case. We excluded those cases in which anal basal pressure was measured longer than 12 months before the botulinum toxin injection or more than 3 months afterwards. We also excluded the cases of patients who received anal surgery, which may influence anal function, between the botulinum toxin injection and manometry (Figure 1). This study was performed in accordance with the Ethical standards of our Institutional Research Committee and registered as M19.235067.

### Treatment Procedure

All the patients referred for botulinum toxin therapy for chronic constipation were given injections in the anal sphincter according to the same protocol. We administered the injection with the patient in supine position. The patients were treated under general anesthesia, without locoregional anesthesia. A rectal speculum was used for clear vision. We administered the same dose of 100 IU of botulinum toxin (Botox, Allergen, the Netherlands) with each injection. We inserted a 27-guage needle into the anal sphincter parallel to the anal canal axis by penetrating the skin outside the anal verge and injected the botulinum toxin in four quadrants, at 3, 6, 9, 12 o'clock (Supplementary Figure 1). The injections were all administered by the same group of experienced pediatric surgeons.

The laxative treatments that had already been initiated before referring the patients for botulinum toxin therapy were continued for approximately 6 months after administering the botulinum toxin injections. In case patients were treated with rectal washouts before the botulinum toxin treatment, they used NaCl 0.9% once or twice a day. This procedure was continued during the first week after injection. The volume of NaCl was adjusted according to patients' age and weight. After the first week, depending on the severity of the constipation symptoms at that moment, the frequency of rectal washouts was gradually reduced. Instead of following a standardized protocol for the gradual reduction of bowel management, reduction of washouts was strictly personalized in that it was based on each patient's individual symptoms.

### Measuring Anal Basal Pressure

We performed the anal basal pressure test within at most 12 months before the injection and within 3 months after the injection, thereby considering the 3–6 months' effect duration of botulinum toxin (15, 20, 21). We measured anal basal pressure with a Laborie/Unisensor K12981solid-state (Boston type) circumferential catheter (Laborie Portsmouth, NH, USA) with an outer diameter of 12F (Supplementary Figure 1C). After placing the catheter, time was allowed for the child to become used to the catheter and for us to measure the resting pressure correctly. We defined a change in anal basal pressure as the value after injection minus the value before injection. We used the gastrointestinal, high-resolution manometry system Solar GI HRAM, Version 8.23 (Laborie/Medical Measurement Systems, Enschede, the Netherlands) to record and analyse the data. The measurements were performed without anesthesia.

### Evaluation of Symptomatic Improvement

We based symptomatic improvement on the interviews held by medical specialists with the pediatric patients' parents during postoperative consultations. Symptomatic improvement was achieved if parents reported that their children were able to defecate without bowel management and/or without pain and/or effort after receiving botulinum toxin therapy. For analysis, the cut-off value of a 30% decrease in anal basal pressure after injection was considered as effective. We based this value on the report by Minkes and Langer (6).

### Statistical Analysis

All statistical analyses were performed using IBM SPSS Statistics, Version 23.0 (IBM Corp, Armonk, NY, USA). The continuous variables were reported as means ± standard deviations and compared with *t*-tests. Relation between categorical variables was analyzed with Fisher exact test. The Pearson test was used to analyse the correlation between the basal pressure changes after injecting botulinum toxin and other continuous variables. Univariable analysis was used to search for possible predictors of the change in the basal pressure. The multivariable linear regression analysis was used to adjust for any possible cofactors and to find independent factors that may predict the change in basal pressure. The receiver operating characteristic (ROC) curve and Youden index were utilized to determine the optimal cut-off value. A *P*-value <0.05 was accepted as significant. Figures were generated using GraphPad Prism 8.2.0 (GraphPad Software Inc, San Diego, CA, USA).

## Results

### Demographics and Clinical Characteristics

We included 43 pediatric patients who were subjected to botulinum toxin treatment for chronic constipation, 35 (81%) of whom received 1 injection, 7 (16%) received 2 injections and 1 patient (2%) received 3 injections, which resulted in 52 cases (Figure 1). In 30 cases (57.7%) the patients suffered idiopathic constipation, that is they had no organic disorders that could be associated with chronic constipation. In 20 cases (38.5%) the patients had Hirschsprung disease and in 2 (3.8%) cases the patients had congenital anorectal malformation. For the main analysis we included the cases with idiopathic constipation, and for the subanalysis we included the patients with organic disorders. The clinical characteristics of the cases included in this study are summarized in Table 1.

**Table 1:** Clinical characteristics of cases included in this study.

**Variables**	**Idiopathic constipation**	**Organic disorder**
Total number of cases:	30	22
Age (months; median)	20.5 (7.75–53.25)	29.5 (20.5–108.5)
Sex (boys/girls)	19/11 (63%/37%)	15/7 (68%/32%)
Weight (kg; median)	11.1 (8.39–17.03)	13.55 (11.58–27.95)
Rectal washout		
Yes	9 (30%)	15 (68%)
No	21 (70%)	7 (32%)
Time between pre-treatment manometry and injection (days)	101.47 ± 61.81	68.23 ± 60.85
Time interval between the injection and manometry after injection (days)	21.3 ±10	27.68 ±15.26
Number of patients		
Single injection	25 (93%)	10 (63%)
Two injections	1 (4%)	6 (38%)
Three injections	1 (4%)	0

### The Change in Anal Basal Pressure After Botulinum Toxin Therapy in Patients With Idiopathic Constipation

**Figure 2:**
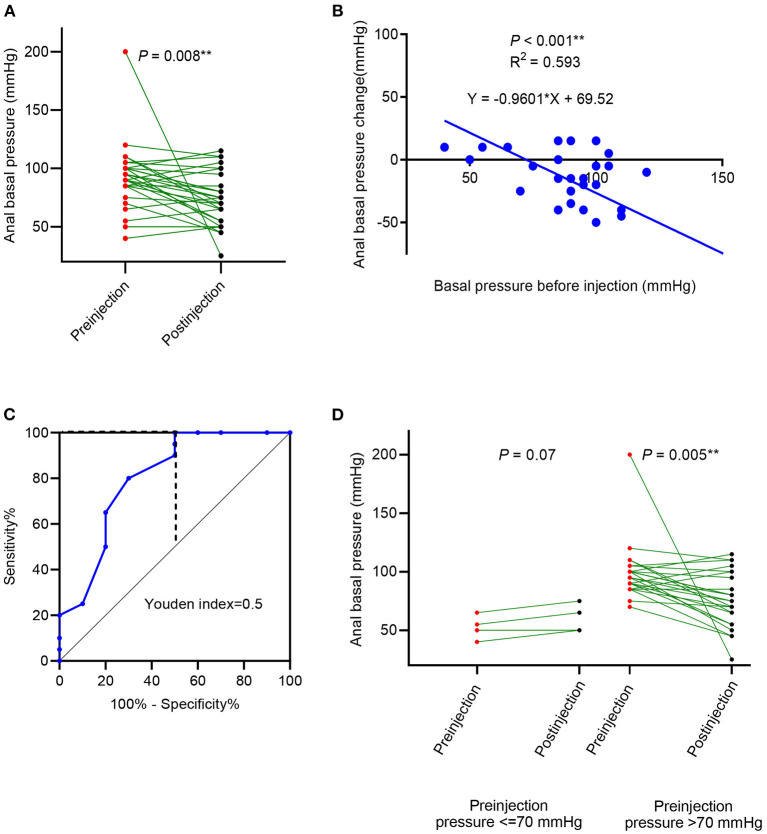
Anal basal pressure change, after botulinum toxin injection in patients with idiopathic constipation. **(A)** The change in anal basal pressure after the botulinum toxin; **(B)** linear correlation between preinjection anal basal pressure and change in anal basal pressure; **(C)** receiver operating characteristic (ROC) curve using preinjection anal basal pressure to predict the decrease of anal basal pressure; **(D)** comparison of anal basal pressure before and after the botulinum toxin injection in patients whose anal basal pressure before injection was ≤ 70 mmHg and >70 mmHg.

In cases with idiopathic constipation, the anal canal basal pressure was 91.33 ± 28.25 mmHg before the botulinum toxin injection and 73.17 ± 22.49 mmHg after the injection (*P* = 0.008, Figure 2A, Table 2). The time between the injection and measurement after injection was 21.3 ± 10 days. In 20 cases (66.7%), anal basal pressure after injecting botulinum toxin had decreased when compared to the pressure before the injection. A graphical representation of the anal basal pressure change is presented as a 2D map in Supplementary Figure 2. No changes were observed in 3 (10%) cases, while pressure increased in 7 cases (23.3%). In these 7 cases the mean value of anal basal pressure before injection was significantly lower than in the other 20 cases whose anal basal pressure decreased after injection (77.14 ± 24.3 mmHg vs. 100.75 ± 26.12 mmHg, *P* = 0.047) (Table 2).

**Table 2:** Anal basal pressure before and after botulinum toxin injection.

**Patients with idiopathic constipation**				
**Variables**	**Number of cases** ***n*** **(%)**	**Basal pressure before injection**^†^ **(mmHg)**	***P*** ^*^	**Basal pressure after injection**^†^ **(mmHg)**
Overall	30	91.33 ± 28.25	0.008	73.17 ± 22.49
**Pressure change**				
Decreased	20 (66.7%)	100.75 ± 26.12	0.001	69.5 ± 20.19
Unchanged	3 (10%)	61.67 ± 20.21	-	61.67 ± 20.21
Increased	7 (23.3%)	77.14 ± 24.3	<0.001	88.57 ± 25.12
**Sex**				
Boys	19 (63.3%)	90 ± 16.83	0.045	81.05 ± 17.04
Girls	11 (36.7%)	93.64 ± 42.37	0.05	59.55 ± 24.95
**Rectal washout**				
Yes	9 (30%)	90 ± 45	0.165	60.56 ± 19.44
No	21 (70%)	91.90 ± 18.61	0.005	78.57 ± 21.92
**Patients with organic disorder**				
Overall	22	88.86 ± 15.81	<0.001	73.64 ± 17.54
**Pressure change**				
Decreased	19 (86.4%)	91.05 ± 15.33	<0.001	72.11 ± 17.82
Unchanged	0	-	-	-
Increased	3 (13.6%)	75 ± 13.23	0.038	83.33 ± 14.43
**Sex**				
Boys	15 (68.2%)	91 ± 17.03	0.002	73.67 ± 18.94
Girls	7 (31.8%)	84.29 ± 12.72	0.094	73.57 ± 15.47
**Rectal washout**				
Yes	15 (68.2%)	85.67 ± 14.86	0.001	67 ± 13.73
No	7 (31.8%)	95.71 ± 16.69	0.091	87.86 ± 17.04

† *Data are presented as mean ± standard deviation*.

P* *, paired t-test between anal basal pressure before injection and anal basal pressure after injection*.

Anal basal pressure before injection and the changes in pressure observed after injection were significantly correlated (*R*^2^ = 0.593, *P* < 0.001, Figure 2B). Using the ROC curve analysis, we found that the preinjection anal basal pressure was 67.5 mmHg when reaching the highest Youden index of 0.50 (Figure 2C). When we rounded off the cut-off value to 70 mmHg, we observed that sensitivity was 100%, specificity was 50%, the positive prediction value was 80% and the negative prediction value was 100%. Consequently, we used 70 mmHg as a cut-off value to distinguish two subgroups: cases with anal basal pressure ≤ 70 mmHg and cases whose anal basal pressure was >70 mmHg. Anal basal pressure decreased significantly after injection in cases whose anal basal pressure before injection was >70 mmHg (*P* = 0.005, Figure 2D). In contrast, in cases whose anal basal pressure before injection was ≤ 70 mmHg, we observed no significant decrease of anal basal pressure (*P* = 0.07, Figure 2D).

### Factors That May Influence Changes in Anal Basal Pressure in Patients With Idiopathic Constipation After the Botulinum Toxin Injection

Using the univariable linear regression analysis we found that in cases with idiopathic constipation the change in anal basal pressure was negatively associated with basal pressure before injection (β = −0.960, *P* < 0.001, Table 3). The change in anal basal pressure was not significantly associated with age at the time of injection (β = 0.179, *P* = 0.163), the time interval between injection and testing postinjection anal basal pressure (β = 0.441, *P* = 0.510), weight (β = 0.652, *P* = 0.177), number of injections (β =13.293, *P* = 0.387), rectal washouts (β = −16.111, *P* = 0.258) and sex (β = 25.144, *P* = 0.058).

**Table 3:** Univariable linear regression analyses of factors that could influence changes in anal basal pressure change in cases with idiopathic constipation (*n* = 30).

**Univariable analysis**					
**Independent variables**	**Beta coefficient^†^**	**95% CI**		**Standard error**	***P***
		**Lower bound**	**Upper bound**		
Basal pressure before injection (mmHg)	−0.960	−1.268	−0.652	0.150	<0.001
Age at injection^§^ (months)	0.179	−0.077	0.435	0.125	0.163
Time interval (days)^¶^	0.441	−0.912	1.793	0.660	0.510
Weight (kg)	0.652	−0.313	1.617	0.471	0.177
Number of injections	13.293	−17.686	44.271	15.123	0.387
**Rectal washout**
Yes	−16.111	−44.691	12.469	13.952	0.258
No	0^‡^				
**Sex**
Boys	25.144	−0.916	51.203	12.722	0.058
Girls	0^‡^				
**Multivariable analysis**
Basal pressure before injection (mmHg)	−0.913	−1.153	−0.672	0.117	<0.001
Weight (kg)	0.512	0.011	1.013	0.243	0.046
Rectal washout	−21.015	−35.598	−6.432	7.081	0.007
**Sex**
Boys	22.971	9.205	36.736	6.684	0.002
Girls	0^‡^				

† *Unstandardised beta coefficient*;

‡ *Reference category*.

§ *Age and weight were significantly correlated and therefore, for multivariable analysis, age was not taken as a cofactor*.

¶ *Time interval between injection and anal basal pressure measurement after injection*.

Weight was significantly correlated with the age (*r* = 0.951, *P* < 0.001). When analyzing correlation of anal basal pressure changes with weight and with age using Pearson' correlation, we found that the r and *P-*values were comparable for age and weight (*r* = 0.261, *P* = 0.163 and r = 0.253, *P* = 0.177, respectively). Because weight can differ between patients of the same age and based on our clinical experience, we think that it is weight itself rather than age that contributes to the efficacy of botulinum toxin therapy. Therefore, to investigate the predictive value of anal basal pressure before injection for anal basal pressure change, we chose to adjust only for weight in the multivariable analysis. The four variables included in the multivariate analysis, that is anal basal pressure before injection, rectal washout and weight and sex, were independent of each other (Supplementary Table 1). Using multivariable analysis, we found that the anal basal pressure change after injection was negatively associated with anal basal pressure before injection (β = −0.913, *P* < 0.001) and rectal washout (β = −21.015, *P* = 0.007). Furthermore, change in anal basal pressure was positively associated with weight (β = 0.512, *P* = 0.046) and with being a boy (β = 22.971, *P* = 0.002) (Table 3).

### Clinical Symptomatic Improvement in Patients With Idiopathic Constipation

**Figure 3:**
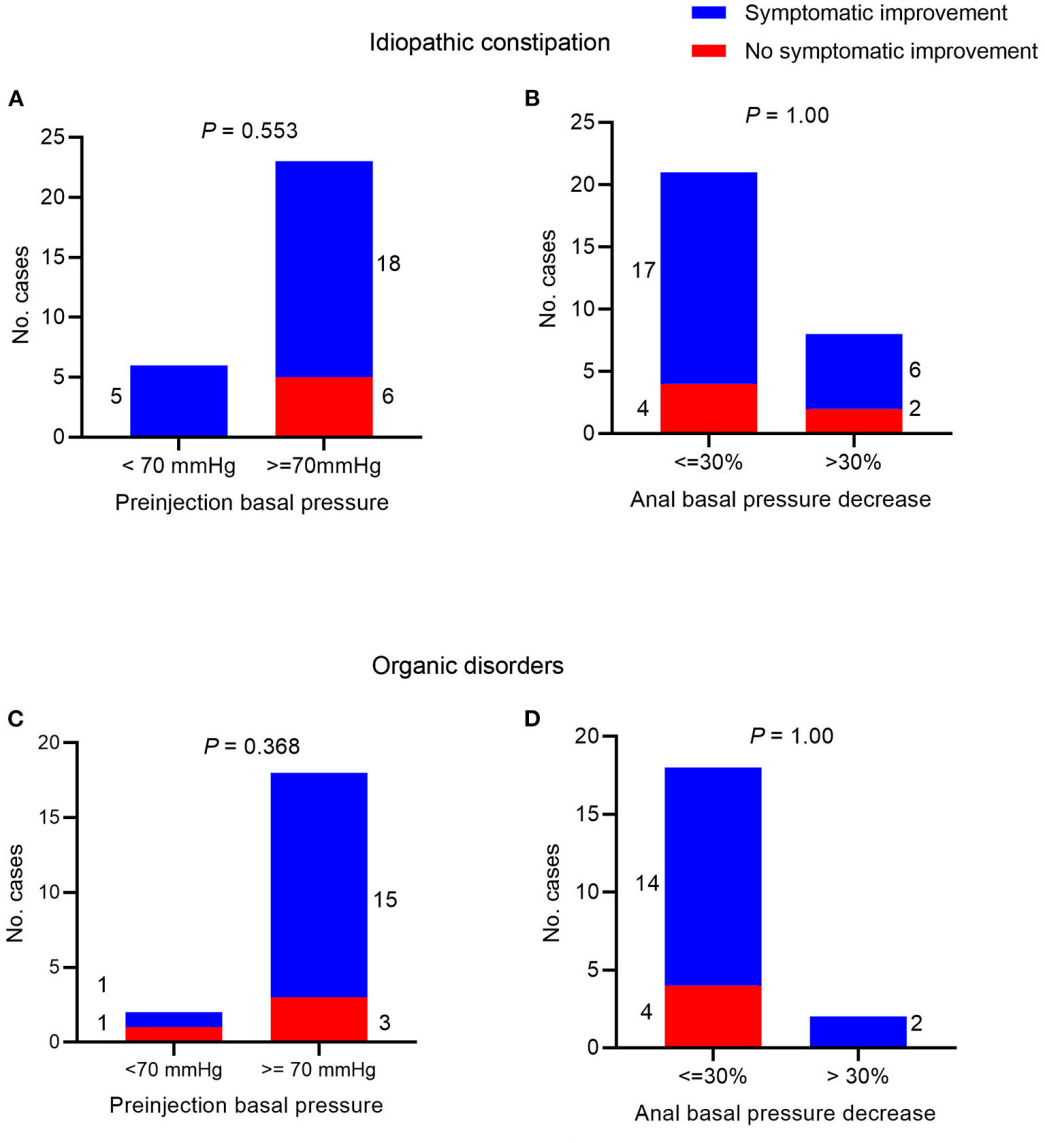
Symptomatic improvement after botulinum toxin injection. In patients with idiopathic constipation and Chi-square test between symptom improvement and **(A)** preinjection anal basal pressure <70 mmHg/≥70 mmHg; **(B)** decrease of anal basal pressure ≤ 30/>30% post-injection. In patients with organic disorders and Chi-square test between symptom improvement and **(C)** preinjection anal basal pressure <70 mmHg/≥70 mmHg; **(D)** decrease of anal basal pressure ≤ 30%/>30% post-injection.

Information regarding symptomatic improvement was available in 29 cases of patients with idiopathic constipation. We found that 23 (79%) cases experienced symptom improvement and that the symptomatic improvement was associated with neither preinjection anal basal pressure nor decrease of the anal basal pressure (*P* = 0.553 and 1.00, respectively, Figure 3).

### The Change in Anal Basal Pressure After Botulinum Toxin Injection in Patients With Organic Constipation

**Figure 4:**
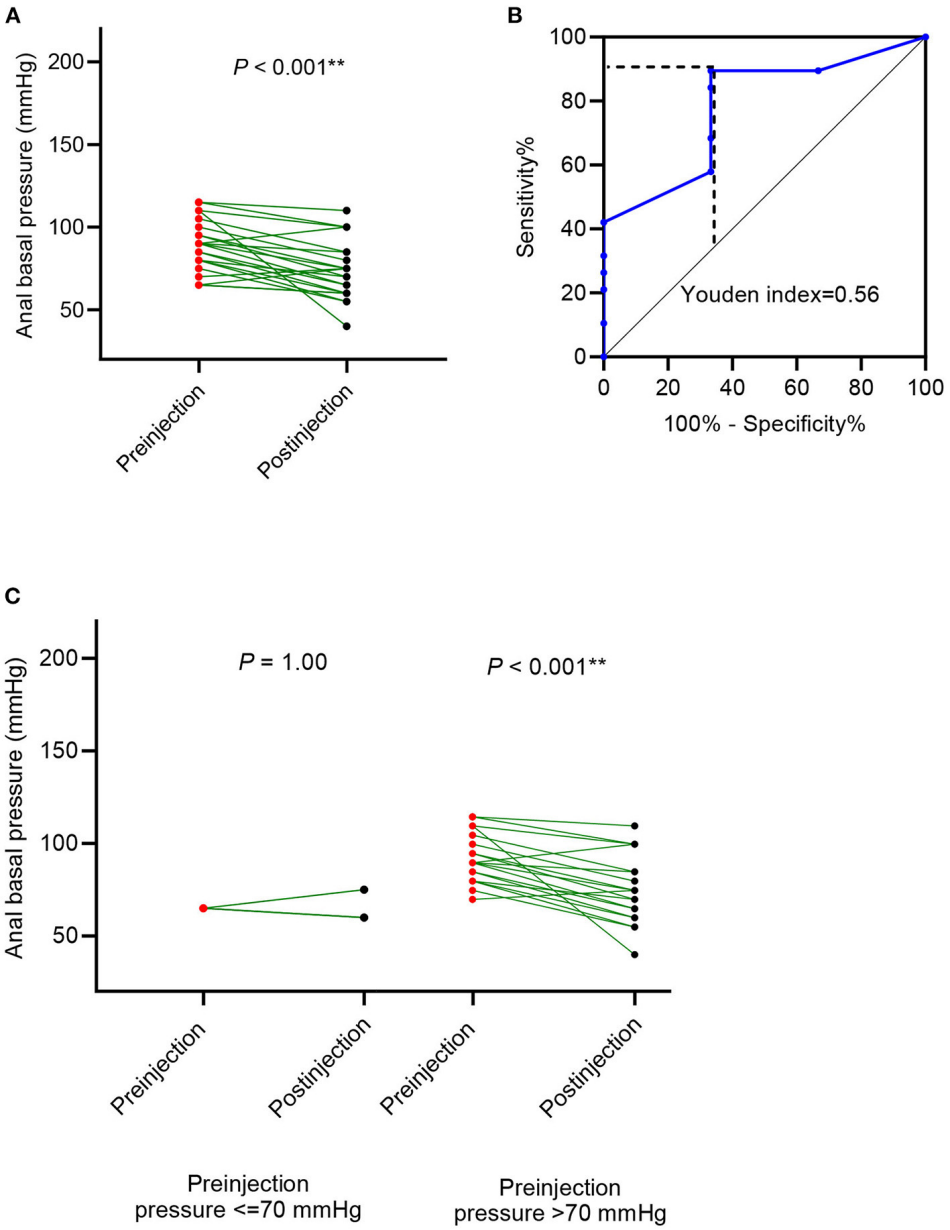
Anal basal pressure change after botulinum toxin injection in patients with organic disorders, such as Hirschsprung's disease (HD) and congenital anorectal malformations (CARM). **(A)** The change in anal basal pressure after botulinum toxin injection; **(B)** receiver operating characteristic (ROC) curve using preinjection anal basal pressure to predict the decrease of anal basal pressure decrease; **(C)** comparison of anal basal pressure before and after the botulinum toxin injection in patients whose anal basal pressure before injection was ≤ 70 mmHg and >70 mmHg.

In cases with organic constipation, the anal canal basal pressure was 88.86 ± 15.81 mmHg before the botulinum toxin injection and 73.64 ± 17.54 mmHg after the injection (*P* < 0.001 Figure 4A, Table 2). The time between the injection and measurement after injection was 27.68 ± 15.26 days. In 19 cases (86.4%), anal basal pressure after injecting botulinum toxin had decreased when compared to the pressure before the injection. No change was observed in none of the cases. Pressure increased in 3 cases (13.6%). In these 3 cases, the mean value of anal basal pressure before injection was lower than in the other 19 cases whose anal basal pressure decreased after injection. The difference, however, was not statistically significant (75 ± 13.23 mmHg vs. 91.05 ± 15.33 mmHg, *P* = 0.103).

Anal basal pressure before injection and the change in pressure observed after injection were correlated (*R*^2^ =0.167, *P* = 0.059). Using the ROC curve analysis, we found that preinjection anal basal pressure was 72.5 mmHg when reaching the highest Youden index of 0.562 (Figure 4B). When we rounded off the cut-off value to 70 mmHg, we observed that sensitivity was 89.5%, specificity was 33.3%, the positive prediction value was 89.47% and the negative prediction value was 33.3%. Consequently, we used 70 mmHg as a cut-off value to distinguish two subgroups: cases with anal basal pressure ≤ 70 mmHg and cases whose anal basal pressure was >70 mmHg. Anal basal pressure decreased significantly after injection in cases whose anal basal pressure before injection was >70 mmHg (*P* < 0.001, Figure 4C). In contrast, in cases whose anal basal pressure before injection was ≤ 70 mmHg, we observed no significant decrease of anal basal pressure (*P* = 1.00, Figure 4C).

### Factors That May Influence Change in Anal Basal Pressure After the Botulinum Toxin Injection in Cases With Organic Disorders

Using univariable linear regression analysis we found that also in cases with organic disorders the change in anal basal pressure was negatively associated with basal pressure before injection (β = −0.425, *P* = 0.059). The change in anal basal pressure was not significantly associated with age at the time of the injection (β = 0.058, *P* = 0.488), the time interval between injection and testing postinjection anal basal pressure (β = 0.048, *P* = 0.845), weight (β = 0.157, *P* = 0.671), number of injections (β =-6.563, *P* = 0.418), rectal washout (β = −10.81, *P* = 0.155) and sex (β = −6.619, *P* = 0.392).

Using multivariable analysis, we found that the anal basal pressure change after injection was negatively associated with anal basal pressure before injection (β = −0.577, *P* = 0.009), and rectal washout (β = −16.607, *P* = 0.02), but it was not statistically associated with weight (Table 4).

**Table 4:** Univariable linear regression analyses of factors which possibly influence changes in the anal basal pressure change in cases with organic disorders (*n* = 22).

**Univariable analysis**				
**Independent variables**	**Beta coefficient^†^**	**95% CI**		**Standard error**	***P***
		**Lower bound**	**Upper bound**		
Basal pressure before injection (mmHg)	−0.425	−0.868	0.017	0.212	0.059
Age at injection^§^ (months)	0.058	−0.112	0.228	0.082	0.488
Time interval (days)^¶^	0.048	−0.454	0.550	0.241	0.845
Weight (kg)	0.157	−0.601	0.914	0.363	0.671
Number of injections	−6.563	−23.101	9.976	7.928	0.418
**Rectal washout**					
Yes	−10.810	−26.081	4.462	7.321	0.155
No	0^‡^				
**Sex**					
Boys	−6.619	−22.402	9.164	7.566	0.392
Girls	0^‡^				
**Multivariable analysis**					
Basal pressure before injection (mmHg)	−0.577	−0.990	−0.164	0.197	0.009
Rectal washout	−16.607	−30.310	−2.903	6.547	0.02

† *Unstandardised beta coefficient*;

‡ *Reference category*.

§ *Age and weight were significantly correlated and therefore, for multivariable analysis, age was not taken as a cofactor*.

¶ *Time interval between injection and anal basal pressure measurement after injection*.

### Clinical Symptomatic Improvement in Patients With Organic Disorders Constipation

Information regarding symptomatic improvement was available for 20 cases with organic disorders constipation. We found that 16 (80%) cases experienced symptom improvement and that symptomatic improvement was associated with neither preinjection anal basal pressure nor decrease of the anal basal pressure (*P* = 0.368 and 1.00, respectively, Figures 3C,D).

## Discussion

With this study we demonstrated that preinjection anal basal pressure and rectal washouts contribute to the decrease of anal basal pressure after botulinum toxin therapy. In patients with idiopathic constipation, weight and sex were also associated with the changes in anal basal pressure after treatment with botulinum toxin.

It is known that approximately 66% of patients treated with botulinum toxin for chronic constipation do not respond or show a suboptimal response to this treatment, but the reasons for this observation remain unclear (16). Although botulinum toxin therapy in patients with chronic constipation is intended to decrease the pathophysiologically elevated anal basal pressure, patients are usually referred for treatment without first measuring the pressure. Chumpitazi et al., suggested measuring anal basal pressure before the botulinum toxin injection (18). They proposed that only patients with high anal canal pressure would benefit from botulinum toxin therapy for functional obstruction. They did not, however, provide the cut-off value. Consequently, in clinical practice the definition for high pressure is a subjective matter. Our finding that anal basal pressure had only decreased significantly in patients whose anal basal pressure before injection of botulinum toxin was higher than approximately 70 mmHg, provides such a cut-off value. This finding is supported by the fact that the change in anal basal pressure after injection of botulinum toxin showed a negative linear correlation with the anal basal pressure observed before injection. A ROC analysis also confirmed that sensitivity and specificity regarding prediction of anal basal pressure was highest when the cut-off value was set at 70 mmHg. Interestingly, is seemed that the cut-off value we established could be used independently of the cause of increased anal basal pressure. We base this conclusion on the fact that we found that patients without organic disorders as well as patients with Hirschsprung's disease or patients with anorectal malformations responded to botulinum toxin therapy provided their anal basal pressure was >70 mmHg.

Like Minkes and Langer (6), we too found no relation between changes of anal basal pressure and clinical outcomes in terms of constipation-related symptoms. One might therefore question the value of manometric tests to assess anal basal pressure. With this study, however, we showed that manometry in constipated patients is useful because it can confirm or exclude the presence of increased anal basal pressure—one of the causes of constipation (22). Furthermore, in case of increased anal basal pressure, manometric tests can indicate whether botulinum toxin therapy could be profitable. Nevertheless, manometric assessment should not be considered the only diagnostic tool with which to diagnose the causes of constipation. Moreover, particularly in children, monitoring anal basal pressure both prior to as well as after injecting botulinum toxin may have additional value. It provides a quantitative and objective outcome, viz. the magnitude of decrease of anal basal pressure, which can be used to follow the patient. Currently, treatment efficacy is based mostly on symptomatic improvement, which is extremely subjective, especially in case of young pediatric patients. A recent systematic review by Roorda et al., showed that the prevalence of symptomatic improvement of pediatric patients varies between 17 to 91% (16). Such a wide range might result from the fact that the youngest patients often cannot describe their symptoms themselves, while their parents are unable to provide objective information concerning the severity of certain symptoms.

Even though we were unable to pinpoint the exact reason why decreased anal basal pressure was not associated with symptomatic improvement in our study, we think it might be due to one or more of the following reasons. First, for this study the information regarding the symptoms was neither collected objectively nor investigated systematically. This was a result of the retrospective study design and because at our hospital medical specialists do not use a validated tool routinely during control visits. Second, although increased anal basal pressure is frequently considered to be the sole cause of constipation, other causes might coexist with increased pressure and, as a consequence reducing anal basal pressure alone may not be sufficient to lead to symptomatic improvement. There are also causes that could lead to constipation independent of anal basal pressure, for instance, decreased colon motility (23), in which case decreased anal basal pressure will also not result in symptomatic improvement. Finally, the reason that symptoms persist in congenital cases such as Hirschsprung's disease may reside in retained aganglionic segments or other dysganglionoses in the proximal colon, events that may explain postoperative enterocolitis, but constipation as well (24, 25). A noteworthy finding by Meunier et al., is that among children with chronic constipation over 50% have low or normal anal basal pressure (12). This information, taken together with our finding, explains why not all patients respond positively to botulinum toxin therapy. Apparently, anal basal pressure needs to be sufficiently elevated in order to decrease after botulinum toxin injection. This finding indicates the importance of monitoring anal basal pressure prior to injecting botulinum toxin. However, currently such monitoring does not occur frequently according to the literature. Perhaps this is because anorectal manometry is not routinely available at all hospitals where botulinum toxin injections are administered. Moreover, the current criteria for referring a patient for botulinum toxin therapy has been undefined on account of the lack of a cut-off value. Thus, little could be gained by measuring the pressure before the injection. Now, the insight that injecting botulinum toxin into the anal sphincter when its pressure is lower than 70 mmHg does not decrease anal basal pressure, provides a clear indication which patients should not be referred for this treatment if the aim is to decrease anal basal pressure. Noteworthy, this value is valid only if the anal basal pressure is measured with a solid-state catheter. For other systems, such as a water perfused system, follow-up studies should be performed to determine such a cut-off value. Our finding partially corroborates the study of Lindsay et al., who also found an association between the response of anal sphincter to botulinum toxin and anal basal pressure before the botulinum toxin injection (26).

In the group of patients with idiopathic constipation we found that patients' ages and weights both correlated with the changes in basal pressure. It is natural that as children grow older, their weight and musculature increases. Weight, however, may vary greatly even between the children of the same age. In clinical practice a medical specialist should therefore adjust the dose according to children's weights rather than to their ages. For the same dosage of botulinum toxin, the higher the child's weight, the less obvious the decrease in anal basal pressure. Moreover, male sex seems to contribute negatively to treatment outcomes when measured in terms of decrease in anal basal pressure. We explain this observation by the fact that male patients usually have a longer anal canal and that the volume of their anal sphincter muscle is larger (27). We did not confirm the correlation between the change in basal pressure and either age or weight in the group with organic constipation. This might have been caused by the smaller size of this group.

In our study the time interval between injection and the postinjection test was not correlated with changes in anal basal pressure. According to the literature, the effect duration of botulinum toxin varies between 3 to 6 months. To avoid any bias caused by the diminishing effect of botulinum toxin, we excluded all the cases with a time interval of more than 3 months between injection and the postinjection test. Additionally, we took the time interval between the injection and the postinjection anal basal pressure measurement as a cofactor in regression analysis to correct for it statistically (15, 20, 21).

Finally, we demonstrated that rectal washouts also contribute significantly to the magnitude of decrease of anal basal pressure after botulinum toxin therapy. This finding is corroborated by the study of Chan et al., who showed that rectal washouts are an useful tool to manage chronic constipation in adults (9). Some physicians consider rectal washouts to be relatively invasive in the sense that repeated washouts might irritate the rectum, damage the mucosa (28) and increase anal canal pressure and that, consequently, resulting in even more severe constipation. Our study showed that such concerns are unjustified. Regarding the relation between bowel washouts and the decrease of anal basal pressure, we think that this relation might be caused by the fact that there are receptors of the anal external sphincter continence reflex (AESCR) in the mucosa of anal canal (29). In healthy subjects the AESCR controls fecal continence by involuntary contraction of the external anal sphincter. In constipated patients, hard feces passing the anal canal during defecation may irritate and damage the mucosa of the anal canal and make the AESCR receptors hypersensitive. In turn, this could lead to overactivation of the AESCR, causing the spasm of the anal sphincter, and thus lead to chronically increased anal basal pressure. We have described this hypothesis before (30). Bowel management softens the feces. This supports regeneration of anal mucosa and prevents it from damaging, which stops the AESCR from overreacting, and allows the anal basal pressure to decrease to its physiological level.

This study has several limitations. First, on account of its retrospective design, some information such as the frequency and duration of rectal washouts, is missing. Second, we were unable to analyse detailed symptomatic improvement in order to compare it to physiological changes. It is not a standard procedure in our hospital to use validated tools to assess constipation symptoms during control visits, which in case of this retrospective study disabled consistent collection of outcomes.

## Conclusion

In summary, botulinum toxin therapy significantly decreases anal basal pressure when the preinjection pressure is higher than 70 mmHg. In our opinion, patients suffering from severely elevated anal canal pressure should be advised to use rectal washouts in combination with botulinum toxin therapy to increase treatment efficacy.

## Data Availability Statement

The datasets presented in this article are not readily available because of ethical concerns, the data has potentially identifiable information. Requests to access the datasets should be directed to corresponding author.

## Ethics Statement

The studies involving human participants were reviewed and approved by the Ethical Committee of University Medical Center Groningen. Written informed consent to participate in this study was not required by the Ethical Committee.

## Author Contributions

GS: conducting the study, collecting, analyzing interpreting data, and drafting the manuscript. MT: conceptual design of the study, interpreting data, and critical revision of the manuscript. PB: conceptual design of the study, interpreting data, and critical revision of the manuscript. All authors contributed to the article and approved submitted version.

## Funding

GS contribution to this study was supported by a joint fellowship from the University Medical Center Groningen and the China Scholarship Council (Grant number CSC 2018 0831 0226 to GS).

## Conflict of Interest

The authors declare that the research was conducted in the absence of any commercial or financial relationships that could be construed as a potential conflict of interest.

## Publisher's Note

All claims expressed in this article are solely those of the authors and do not necessarily represent those of their affiliated organizations, or those of the publisher, the editors and the reviewers. Any product that may be evaluated in this article, or claim that may be made by its manufacturer, is not guaranteed or endorsed by the publisher.

## Supplementary Material

Supplementary Figure 1 The administration of the botulinum toxin injections. **(A)** Injections were administered in four quadrants; **(B)** injection migratory direction and distance; **(C)** measurement of anal canal pressure with manometry.

Supplementary Figure 2 The 2D map representing graphical outcome of anal canal pressure measured with manometry from one patient. **(A)** Before injection, the anal canal pressure was high; **(B)** after injection anal canal pressure has decreased.

Supplementary Table 1 Correlation analysis^†^ between the variables taken into the multivariable linear regression analysis in cases with idiopathic constipation (n=30).
