# Supplementary figures and images for: High Anal Canal Pressure and Rectal Washouts Contribute to the Decrease of Anal Basal Pressure After Botulinum Toxin Injections in Paediatric Patients With Chronic Constipation
Source: Front Pediatr. 2022 Mar 22;10:819529. doi: 10.3389/fped.2022.819529 (PMC8980778; doi:10.3389/fped.2022.819529)

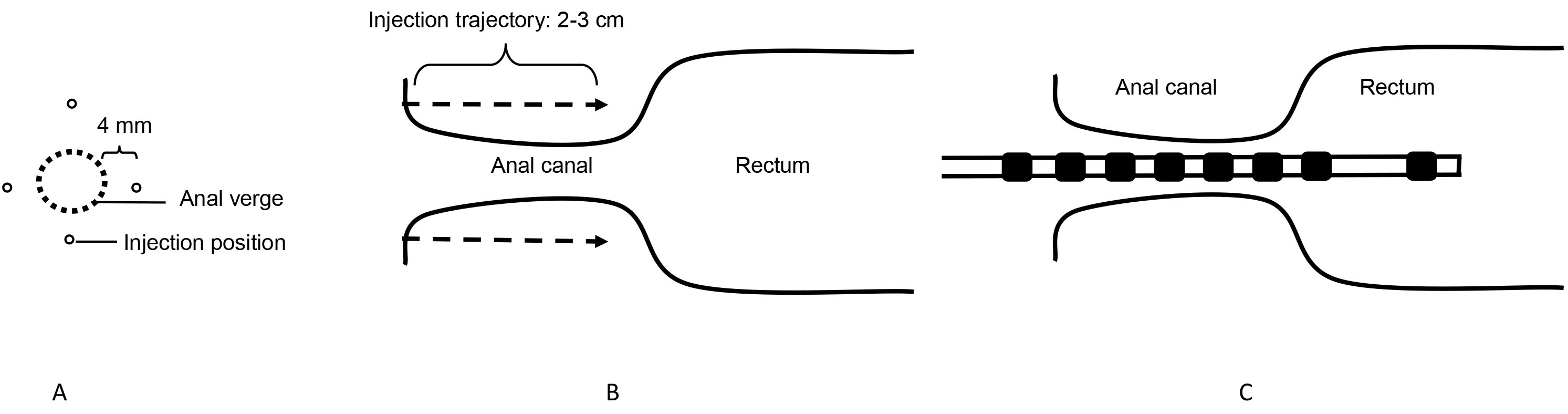

Supplement: Supplementary Figure 1 — The administration of the botulinum toxin injections. (A) Injections were administered in four quadrants; (B) injection migratory direction and distance; (C) measurement of anal canal pressure with manometry. [file Image_1.JPEG]

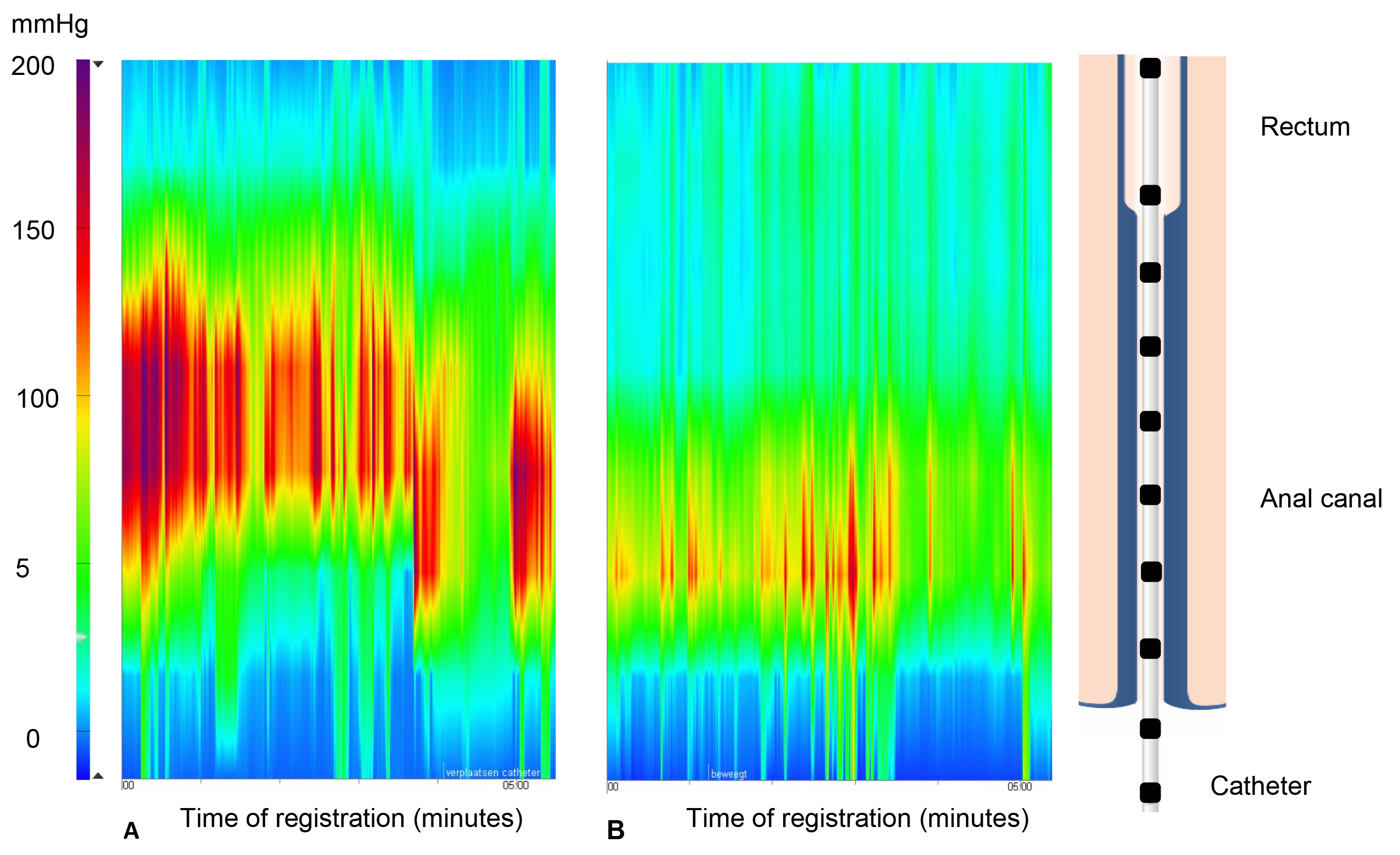

Supplement: Supplementary Figure 2 — The 2D map representing graphical outcome of anal canal pressure measured with manometry from one patient. (A) Before injection, the anal canal pressure was high; (B) after injection anal canal pressure has decreased. [file Image_2.JPEG]
