# Supplementary material for: High Anal Canal Pressure and Rectal Washouts Contribute to the Decrease of Anal Basal Pressure After Botulinum Toxin Injections in Paediatric Patients With Chronic Constipation
Source: Front Pediatr. 2022 Mar 22;10:819529. doi: 10.3389/fped.2022.819529 (PMC8980778; doi:10.3389/fped.2022.819529)
Supplement: Supplementary Table 1 — Correlation analysis† between the variables taken into the multivariable linear regression analysis in cases with idiopathic constipation (n=30). [file Table_1.DOCX]

| **Supplementary Table 1. Correlation analysis**^†^ **between the variables taken into the multivariable linear regression analysis in cases with idiopathic constipation (n=30)** | | | | | |
| --- | --- | --- | --- | --- | --- |
| **Variables** | | **Basal pressure before**  **injection (mmHg)** | **Weight (kg)** | **Rectal washout** | **Sex** |
| **Basal pressure before**  **injection (mmHg)** | r | n.a. | -0.134 | -0.031 | -0.063 |
|  | *P* |  | 0.479 | 0.869 | 0.741 |
| **Weight (kg)** | r | -0.134 | n.a. | 0.138 | -0.017 |
|  | *P* | 0.479 |  | 0.467 | 0.930 |
| **Rectal washout** | r | -0.031 | 0.138 | n.a. | 0.045 |
|  | *P* | 0.869 | 0.467 |  | 0.812 |
| **Sex** | r | -0.063 | -0.017 | 0.045 | n.a. |
|  | *P* | 0.741 | 0.930 | 0.812 |  |

^†^Pearson’s correlation test; n.a. – not applicable
